# Climate change anxiety and pro-environmental behaviours: disentangling gender disparities

**DOI:** 10.3389/fsoc.2025.1589501

**Published:** 2025-06-09

**Authors:** Mariana Pinho

**Affiliations:** ECOMARE, Centre for Environmental and Marine Studies, Department of Biology, University of Aveiro, Aveiro, Portugal

**Keywords:** climate change anxiety, pro-environmental behaviour, gender, environmental identity, climate change perceptions

## Abstract

**Introduction:**

Climate change represents the most significant environmental and social issue of our time. Climate change anxiety has been identified as a relevant consequence of climate change globally.

**Methods:**

The current study explored how climate change anxiety and pro-environmental behaviour vary with gender and social psychological characteristics, using a nationally representative Portuguese sample.

**Results:**

The findings revealed that women reported higher levels of climate change anxiety compared to men, and this was driven by women’s higher levels of climate change anxiety cognitive impairment. Women also indicated more frequent pro-environmental behaviours, higher levels of environmental identity and climate change perceptions than men. The findings further showed similar relations for men and women, between social psychological mechanisms (environmental identity and climate change perceptions) and their impact on climate change anxiety and some types of pro-environmental behaviours. The results also demonstrated that climate change perceptions mediated the effect of environmental identity on pro-environmental behaviours and those mediations were further moderated by gender.

**Discussion:**

The results highlight the importance of exploring the gender gap in environmental related attitudes and behaviours and the incorporation of gender mainstreaming in environmental sustainability policies and programmes.

## 1 Introduction

Climate change represents the most significant environmental and social issue of our time, being recognised as a health emergency. Growing evidence shows that experiencing the effects of climate change (e.g., rising temperatures, extreme weather events) has devastating impacts on physical (e.g., Marselle et al., 2019; Perera and Nadeau, 2022; Rocque et al., 2021) and mental health (e.g., Clayton and Karzsia, 2020; Cosh et al., 2024; Dodds, 2021). A relevant example of such impact is climate change anxiety. Climate change anxiety can be defined as the concern or distress in response to climate change, which can result in psychological and physical symptoms (Clayton, 2020; Dodds, 2021). For example, depression, anxiety, stress or insomnia (Patrick et al., 2023; Schwartz et al., 2023).

Climate change anxiety is a serious social concern, increasingly recognised as a public health issue (Dodds, 2021; Ogunbode et al., 2023). When climate change anxiety reaches high levels, it can turn maladaptive, impairing one’s capacity to properly respond to the climate crisis (Crandon et al., 2024; von Gal et al., 2024).

If the climate crisis is not adequately addressed, it will not only have devastating effects on the population but also affect human rights and exacerbate social inequality globally, disproportionately impacting vulnerable people (e.g., women, people suffering from mental illness, lower income) and healthcare systems (CISL, 2019; IMF, 2021; UN Human Rights Council, 2019).

Recent research suggests that socio-demographic characteristics (such as younger age, left-leaning political ideologies) relate to higher levels of climate change anxiety (e.g., Asgarizadeh et al., 2023; Hickman, 2024; Hickman et al., 2021; Whitmarsh et al., 2022). Despite gender being associated with higher vulnerability to climate change (e.g., Carr et al., 2024; Chen and Yu, 2024; Eastin, 2018; Zeng et al., 2024), research on the relationship between gender, climate change anxiety and pro-environmental behaviour remains mixed. Many studies have found that women are more likely to experience higher levels of climate change anxiety (e.g., Heeren et al., 2022; Hickman et al., 2021; Wullenkord et al., 2021) and engage in pro-environmental actions (e.g., Li et al., 2022; Wang and Li, 2021). Nevertheless, other studies did not find such associations (e.g., Asgarizadeh et al., 2023; Clayton and Karzsia, 2020; Schwartz et al., 2023; Whitmarsh et al., 2022) or found contrary results (e.g., Islam and Managi, 2019; Xiao and Hong, 2010).

To adopt solutions that accelerate meaningful change towards a more inclusive climate policy, it is essential to expand the focus of enquiry from socio-demographic characteristics to the social psychological characteristics that guide climate change anxiety and pro-environmental behaviour.

### 1.1 Gender differences in climate change anxiety

The concept of climate change being gender neutral has been challenged (e.g., Andrijevic et al., 2025; Reggers, 2019). The impacts of climate change must be considered in the gendered sociocultural context within which men and women exist (Arora-Jonsson, 2011; Pickering and Dale, 2023). Despite the considerable progress towards gender equality, women remain more vulnerable to the consequences of climate change and are less likely to survive natural disasters, due to disparities in human rights, decision-making, access to resources, lack of education and financial independence (Andrijevic et al., 2025; Desai and Zhang, 2021; Nellemann et al., 2011). Women are also more likely than men to suffer from different forms of anxiety (see McLean et al., 2011, for a review). However, findings regarding gender differences in experiencing climate change anxiety remain inconsistent. Studies found that women suffer from climate change anxiety and negative emotions related to climate to a higher extent (e.g., Clayton et al., 2023; Coffey et al., 2021; Elert and Lundin, 2022; Sorensen et al., 2018) and express higher levels of climate change risk perception than men (Ergun et al., 2024). This is true even among women who live in wealthier countries and contexts less exposed to climate change (Bush and Clayton, 2023; McCright and Sundström, 2013). Research also shows that women take more responsibility for climate change (Arora-Jonsson, 2011; Desai and Zhang, 2021; Seedat and Rondon, 2021). Nevertheless, other studies did not find any significant gender differences (e.g., Asgarizadeh et al., 2023; Clayton and Karzsia, 2020; Schwartz et al., 2023; Whitmarsh et al., 2022).

### 1.2 Gender disparities in social psychological characteristics and pro-environmental behaviour

Some research shows that climate change anxiety is associated with pro-environmental behaviour, meaning higher levels of climate change anxiety are related to more frequent pro-environmental behaviour and climate action (e.g., Chapman and Peters, 2024; Tam et al., 2023; Whitmarsh et al., 2022). Evidence of the opposite has also been found, namely individuals who experienced moderate levels of climate change anxiety engaged in pro-environment behaviours more frequently than those with either low or high levels of climate change anxiety, indicating the possibility of high levels of climate change anxiety restraining pro-environmental behaviour (Coates et al., 2024, 2025).

Social psychological characteristics, such as higher levels of environmental identity and climate change perceptions, have also been positively associated and predicted more frequent pro-environmental behaviour (e.g., Ajibade and Boateng, 2021; Brügger et al., 2021; van Valkengoed et al., 2024). Climate change perceptions can be defined as the degree to which climate change is believed to be real, caused by humans and having negative consequences (Leiserowitz et al., 2021; Steg, 2023). Climate change perceptions are linked to pro-environmental behaviour and support for mitigation policies (Brink and Wamsler, 2019; Steg, 2023; van Valkengoed et al., 2021). Furthermore, higher levels of climate change perceptions have been associated with a higher likelihood of adopting adaptation behaviours (van Valkengoed et al., 2024). For example, higher climate change perceptions enhance one’s awareness of the effects of climate change and intensify their personal responsibility and moral obligation, consequently motivating their pro-environmental behaviour (e.g., Steg, 2023; Zawadzki et al., 2020). Gender differences have also been found regarding climate change perceptions, with men being more likely to express climate denial than women (e.g., Effrosynidis et al., 2022; McCright and Dunlap, 2011).

Environmental identity refers to the personal connection to a part of the nonhuman natural environment (Clayton, 2003, 2012). A more salient environmental identity has been linked to and predicted an increased engagement in pro-environmental behaviour and stronger efforts to combat climate change (e.g., Ajibade and Boateng, 2021; Young et al., 2020; Whitburn et al., 2020). In one study, gender was found to impact the development of environmental identity through the influence of gender stereotypes (Miao and Cagle, 2020). Other studies have also found gender differences in environmental identity, namely women reporting significantly higher levels of environmental identity than men (e.g., Clayton and Kilinç, 2013; Prévot et al., 2016; Yue et al., 2021).

Similar to climate change anxiety, research on gender differences in pro-environmental behaviour remains mixed. Some research identified gender as a predictor of pro-environmental attitudes and behaviours. Specifically, women engage more in climate-mitigating actions, pro-environmental behaviour and hold greater pro-environmental values than men (Elert and Lundin, 2022; Coffey et al., 2021; Li et al., 2022; Maartensson and Loi, 2021; Pickering and Dale, 2023). However, other studies found no gender differences (e.g., Berenguer et al., 2005; Blocker and Eckberg, 1997) or opposite results (e.g., Islam and Managi, 2019; Xiao and Hong, 2010).

### 1.3 Present study

The current study aims to reveal how climate change anxiety and pro-environmental behaviour vary with gender and social psychological characteristics, namely environmental identity and climate change perceptions. With the lack of diversity and inclusion in sustainability, power structures and inequalities continue to be replicated in climate change discourse, policies and initiatives (Arora-Jonsson, 2011; Bell, 2021). Therefore, understanding the role of gender is essential to prevent the exacerbation of the inequality gap and contribute to the design of effective and equitable environmental policy and mechanisms to address climate change. Research has shown that women experience climate change anxiety more intensely and exhibit less climate change denial than men (e.g., Clayton et al., 2023; Coffey et al., 2021; Effrosynidis et al., 2022). Therefore, it was predicted that women would have higher levels of climate anxiety than men (Hypothesis 1) and that men would have lower climate change perceptions than women, meaning they will be less likely to believe climate change is real, has anthropogenic origins and negative consequences worldwide (Hypothesis 2).

Previous studies additionally found gender differences in environmental identity and pro-environmental behaviour, namely, women report significantly higher levels of environmental identity and pro-environmental behaviour compared to men (e.g., Clayton and Kilinç, 2013; Elert and Lundin, 2022; Prévot et al., 2016). Consequently, it was hypothesised that women would have higher levels of environmental identity (Hypothesis 3) and would exhibit higher pro-environmental behaviour than men (Hypothesis 4).

Finally, according to the whole rationale previously presented, it was hypothesised that climate change perceptions would mediate the effect of environmental identity on climate change anxiety and pro-environmental behaviour (Hypothesis 5). This mediation by climate change perceptions would be moderated by gender (Hypothesis 6). Specifically, associations between environmental identity, climate change anxiety and pro-environmental behaviours mediated by climate change perceptions will be attenuated in men.

Inconsistencies among previous results regarding gender differences in climate change anxiety and pro-environmental behaviour are primarily due to the variation of sampling methods across the studies. With very few exceptions, most of these studies had convenience samples. Therefore, this study extends previous literature by analysing a representative Portuguese sample and accounting for the inconsistent findings regarding gender differences in climate change anxiety and pro-environmental behaviour. As Portugal is among the European countries with the greatest vulnerability to climate change, likely, the prevalence and severity of climate change anxiety among the Portuguese population is proportionally high (Cardoso et al., 2019; Carvalho, 2024; Climate Change Performance Index, 2025). Consequently, understanding the role of gender is crucial to inform the implementation of climate mitigation and prevention actions and minimise its impacts on the population. Additionally, by assessing the operation of social psychological mechanisms in climate change anxiety and pro-environmental behaviour, the current study adds to previous literature that has mainly focused on demographic determinants. Therefore, to test the hypotheses, the following questions were addressed:

- *Research Question 1*: What are the differences in climate change anxiety and pro-environmental behaviour associated with gender?
- *Research Question 2*: Are there gender differences in the socio psychological characteristics that relate and influence climate change anxiety and pro-environmental behaviour?

## 2 Method

### 2.1 Sample and procedure

Data were collected from 3,300 adults (1,583 men and 1,717 women), with ages ranging from 18 to 74 years old (*M* = 46.29, SD = 13.96). Most participants lived in a city (64.4%) and had children (62.3%). Table 1 shows participants’ socio-demographic characteristics. The sample was recruited at random from a panel of nationally representative members of Qdata, one of the main survey companies in Portugal. Stratified random sampling was applied and the sample size was calculated with a margin of error of 2% and a confidence level of 99% of the total Portuguese current population (10,639,726). The sample contained participants from all demographic groups in the same proportions as the whole Portuguese population. Emails were sent to panellists to complete an online questionnaire that took on average 15 min to complete.

**Table 1:** The demographic characteristics of the participants.

Demographic characteristics	Male	Female	Total
Age			
18–34	19.9%	29.6%	24.97%
35–54	38.1%	40.3%	39.24%
55–74	42%	30.2%	35.79%
Education			
Less than high school/secondary education	11.1%	5.8%	8.3%
High school/secondary education diploma	32.9%	28.7%	30.7%
Post-secondary non-higher education	6.9%	4.4%	5.6%
Higher education/academic degree	49.1%	61.1%	55.4%
Marital/relationship status			
Single	26.5%	31.2%	28.9%
Married/civil partnership	65.7%	52.9%	59%
Widower	0.9%	1.9%	1.4%
Divorced/separated	6.9%	14.1%	10.7%
Monthly household income			
≤€590	4.5%	6.5%	5.5%
€590 – €891	8.5%	12.5%	10.5%
€891 – €1,688	29.9%	34.9%	32.5%
€1,688 – €2083	16.1%	16.8%	16.5%
€2083 – €3,071	24.4%	17.4%	20.8%
€3,071 – €6,720	15.5%	10.7%	13%
≥ €6,720	1%	1.3%	1.2%
Professional status			
Student	3.8%	3.2%	5.7%
Employed	73.7%	73.6%	71.5%
Retired	15.5%	8.7%	12%
Unemployed	5.8%	8.6%	7.2%
Homemaker	0.3%	4%	2.2%
Other	0.9%	1.9%	1.4%

### 2.2 Measures

#### 2.2.1 Climate change anxiety

Climate change anxiety as a psychological response to climate change was measured using Clayton and Karzsia (2020) 13-item instrument. The measure is composed of two subscales: (a) cognitive impairment (e.g., “*Thinking about climate change makes it difficult for me to sleep*”) and (b) functional impairment (e.g., “*I have problems balancing my concerns about sustainability with the needs of my family*”). Responses were indicated on a 5-point scale ranging from 1 = *Never* to 5 = *Almost always*. The respondent’s average score on each dimension was computed. Cronbach’s alphas for these dimensions were 0.91 and 0.89, respectively. The average of all 13 items was also calculated to obtain a total climate change anxiety score. Cronbach’s alpha for the overall climate change anxiety scale was 0.94.

#### 2.2.2 Environmental identity

Participants’ environmental identity was assessed using Clayton et al.’s (2021) scale. The measure included 14 items that measure individual differences in a stable sense of interdependence and connectedness with nature. Responses were indicated on a scale from 1 = *Not at all true of me* to 7 = *Completely true of me*. The average score was computed to measure participants’ overall environmental identity. Cronbach’s alpha for this measure was 0.93.

#### 2.2.3 Pro-environmental behaviour

Pro-environmental behaviours were measured using an adapted version of Markle’s (2013) scale. The scale included six conservation behaviours addressing how often individuals reduce their consumption of heating, air-conditioning, hot water, and lighting. Responses were indicated on a scale from 1 = *Never* to 5 = *Always*. The average score was computed to measure participants’ conservation behaviours. Cronbach’s alpha for this measure was 0.69. Two items about decreased consumption of meat (beef, pork, and poultry) and increasing consumption of vegetarian meals within the last year were also included. Cronbach’s alpha for this measure was 0.57. Transportation related behaviours, such as carpooling, taking public transportation, and walking or cycling instead of driving in the past year, were also evaluated through three items. Responses were indicated on a scale from 1 = *Never* to 5 = *Frequently*. The average score was computed to measure participants’ transportation related behaviours. Cronbach’s alpha for this measure was 0.60.

#### 2.2.4 Climate change perceptions

Participants’ perceptions of climate change were assessed using van Valkengoed et al.’s (2021) scale, which included three types of perceptions: (a) reality (e.g., “*I believe that climate change is real*”); (b) causes (e.g., “*Human activities are a major cause of climate change*”); and (c) valence of consequences (e.g., “*Climate change will bring about serious negative consequences*”). Participants used a 7-point Likert-scale from 1 = *Strongly disagree* to 7 = *Strongly agree*. An average of all climate change perception items was also calculated to create a total score. Cronbach’s alpha for this measure was 0.96.

#### 2.2.5 Socio-demographic variables

Participants indicated their age, gender, occupation, level of education and marital status. Participants also reported the total number of children and their individual monthly income on a seven-point scale ranging from 1 (*less than €590*) to 7 (*more than €6,720*).

### 2.3 Analytic strategy

First, descriptive statistics and Pearson correlations were analysed between climate change anxiety, environmental identity, climate change perceptions and three kinds of pro-environmental behaviours, separately for men and women. To test the first four hypotheses, gender differences were explored regarding climate change anxiety, climate change perceptions, environmental identity and pro-environmental behaviours, using independent sample *t*-tests. To further explore gender differences, a series of multiple regression analyses was conducted separately for men and women. In each analysis, a variable pertaining to climate change anxiety and one measure of pro-environmental behaviour was regressed on the set of environmental identity and climate change perceptions measures. To test the moderated mediation of climate change perceptions in the relationship between environmental identity and climate anxiety; and pro-environmental behaviours (Hypotheses 5 and 6), methods developed by Preacher and Hayes were followed (Hayes, 2022; Preacher et al., 2007) for evaluating conditional indirect effects using the bootstrap procedure (Preacher and Hayes, 2004). Moderated mediation analyses were conducted using Hayes’ (2022) PROCESS program with 1,000 bootstrap samples and bias-corrected 95% confidence intervals.

## 3 Results

### 3.1 Preliminary analysis

Means, standard deviations, and Pearson correlations among the socio psychological measures, climate change anxiety and three kinds of pro-environmental behaviour are presented in Table 2. Correlation analyses were conducted on the full sample, separately for men and women. For men and women, climate change anxiety was positively related with use of carpooling or alternative means of transportation (*r* = 0.28, *p* < 0.001; *r* = 0.19, *p* < 0.001, respectively) and with the consumption of vegetarian meals (*r* = 0.20, *ps* < 0.001 for women and men). However, climate change anxiety was only positively correlated with conservation behaviour (e.g., reducing consumption of heating, air-conditioning) for men (*r* = 0.09*, p* < 0.001).

**Table 2:** Means, standard deviations, and correlations among study measures.

Variables	1	2	3	4	5	6	Men	
							*M*	SD
1.Climate change anxiety	*–*	0.09***	0.28***	0.20***	0.23***	0.12****	1.70	0.64
2.Conservation behaviour	0.04	*–*	0.17***	0.22***	0.39***	0.29***	4.18	0.58
3.Transportation	0.19***	0.13***	*–*	0.14***	0.22***	0.11***	2.80	0.97
4.Food consumption	0.20***	0.23***	0.16***	*–*	0.26***	0.18***	1.57	0.49
5.Environmental identity	0.22***	0.34***	0.19***	0.25***	*–*	0.35***	5.59	0.92
6.Climate change perceptions	0.08***	0.20***	0.12***	0.15***	0.26***	*–*	5.59	0.92
Women *M*	1.80	4.24	2.94	1.69	5.73	6.30		
Women SD	0.69	0.56	1.06	0.47	0.87	0.85		

Higher scores on all measures reflect higher levels of the construct. Correlations for men are presented above the diagonal, for women below the diagonal. ***p < 0.001.

Climate change anxiety, for women and men, was also positively related with environmental identity (*r* = 0.22, *p* < 0.001; *r* = 0.23, *p* < 0.001, respectively) and climate change perceptions (*r* = 0.08, *p* < 0.001; *r* = 0.12 *p* < 0.001, respectively).

### 3.2 Gender differences in climate change anxiety, social psychological characteristics, and pro-environmental behaviour

**Figure 1:**
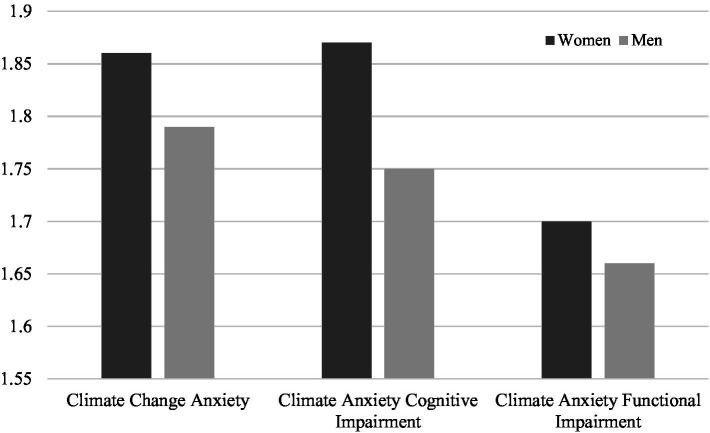
Comparison of mean scores for climate change anxiety between men and women.

The first hypothesis suggested compared to men, women would have higher levels of climate change anxiety. Gender differences in experiencing climate change anxiety were examined using independent sample *t*-tests (see Figure 1). As illustrated in Figure 1, *t*-test comparisons revealed that women (*M* = 1.86, SD = 0.67) reported higher levels of climate change anxiety compared to men (*M* = 1.79, SD = 0.63), (*t* (3243) = −3.21, *p* < *0*.001), confirming the first hypothesis. This was driven by women’s higher levels of climate change anxiety cognitive impairment (*M* = 1.87, SD = 0.72; *M* = 1.75, SD = 0.68, women and men, respectively) (*t* (3298) = −5.03, *p* < 0.001).

**Figure 2:**
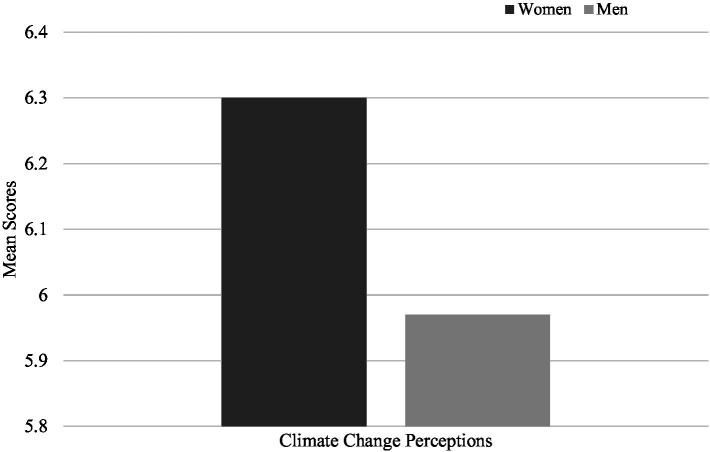
Comparison of mean scores for climate change perceptions between men and women.

**Figure 3:**
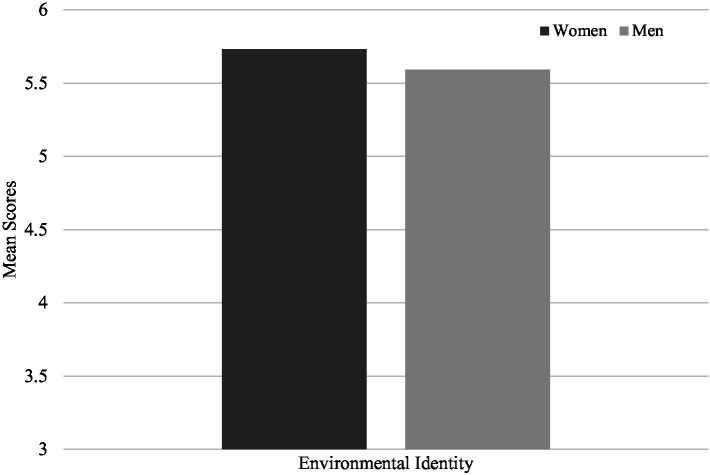
Comparison of mean scores for environmental identity between men and women.

To test the second and third hypotheses, similar analysis were conducted and showed that women indicated higher levels climate change perceptions (*M* = 6.30, SD = 0.85) (Figure 2) and of environmental identity (*M* = 5.73, SD = 0.87) (Figure 3) than men (*M* = 5.97, SD = 1.12, *M* = 5.59, SD = 0.92; respectively) (*t* (3298) = −9.70, *p* < 0.001; *t* (3298) = −4.35, *p* < 0.001), consistent with both hypotheses.

**Figure 4:**
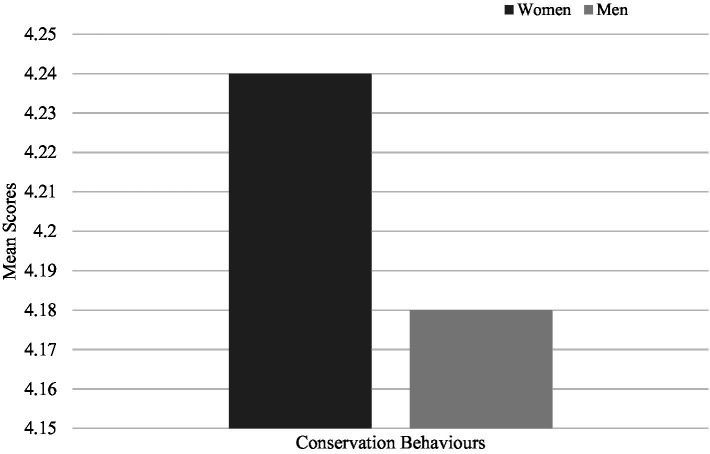
Comparison of mean scores for conservation behaviours between men and women.

**Figure 5:**
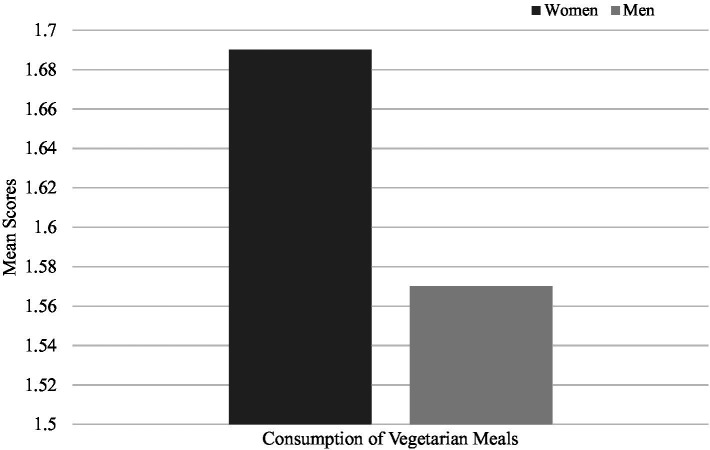
Comparison of mean scores for consumption of vegetarian meals between men and women.

**Figure 6:**
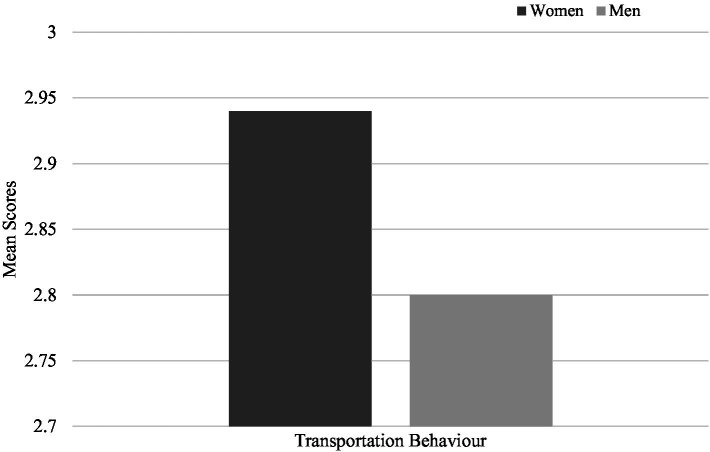
Comparison of mean scores for transportation behaviour between men and women.

Regarding pro-environmental behaviours, women indicated more frequent conservation behaviours (*M* = 4.24, SD = 0.58) compared to men (*M* = 4.18, SD = 0.58), (*t* (2903) = −2.80, *p* = 0.005) (Figure 4), more frequent consumption of vegetarian meals (*M* = 1.69, SD = 0.47*; M* = 1.57, SD = 0.49, women and men respectively), (*t* (3298) = −6.82, *p* < 0.001) (Figure 5) and more frequent use of alternative modes of transportation (*M* = 2.94, SD = 1.06; *M* = 2.80, SD = 0.97, women and men respectively), (*t* (3298) = −3.85, *p* < 0.001) (Figure 6). These results confirm the fourth hypothesis that predicted that women would exhibit higher pro-environmental behaviour than men.

To explore the role of environmental identity and climate change perceptions on climate change anxiety and three types of pro-environmental behaviours, a set of multiple regression analyses was conducted for men and women separately. Table 3 indicates that the regression equation of climate change anxiety on environmental identity was significant and accounted for 5% of the variance for both women and men. Table 3 also shows that regression equations of conservation behaviour and consumption of vegetarian meals on environmental identity were also significant and accounted for 6 to 12% of the variance for women and 7 to 15% for men. Similar results were also found for climate change perceptions. Thus, the more salient environmental identity participants had and the more they believed climate change was real, caused by humans and had negative consequences, the more they reported conservation behaviours and higher consumption of vegetarian meals. However, climate change perceptions were only a significant predictor for the use of alternative modes of transportation by women but not men.

**Table 3:** Multiple regression analyses predicting climate change anxiety and pro-environmental behaviour.

Model	Climate change anxiety		Conservation behaviour		Transportation		Food consumption	
	1	2	1	2	1	2	1	2
a. Women								
Environmental identity	0.22***	0.21***	0.34***	0.31***	0.19***	0.17***	0.25***	0.22***
Climate change perceptions	*–*	0.03	*–*	0.12***	*–*	0.07**	*–*	0.09***
*R* ^2^	0.05***	0.05***	0.12***	0.13***	0.04***	0.04***	0.06***	0.07***
*F*(2, 1,686)		43.64***		112.81***		36.67***		63.52***
b. Men								
Environmental identity	0.23***	0.22***	0.39***	0.33***	0.22***	0.21***	0.26***	0.22***
Climate change perceptions	*–*	0.05	*–*	0.17***	*–*	0.04	*–*	0.10***
*R* ^2^	0.05***	0.05***	0.15***	0.18***	0.05***	0.05***	0.07***	0.07***
*F*(2, 1,557)		45.14***		149.67***		41.63***		64.28***

Standardized beta coefficients are reported. Tests of significance were two-tailed. **p < 0.01; ***p < 0.001.

**Figure 7:**
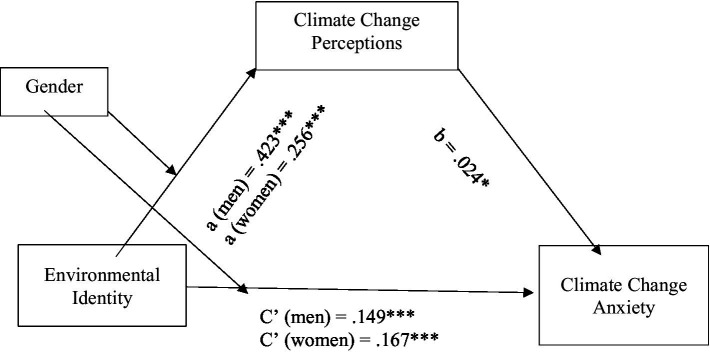
Conditional indirect effects of men’s and women’s environmental identity on climate change anxiety through climate change perceptions. *p < 0.05; ***p < 0.001.

**Figure 8:**
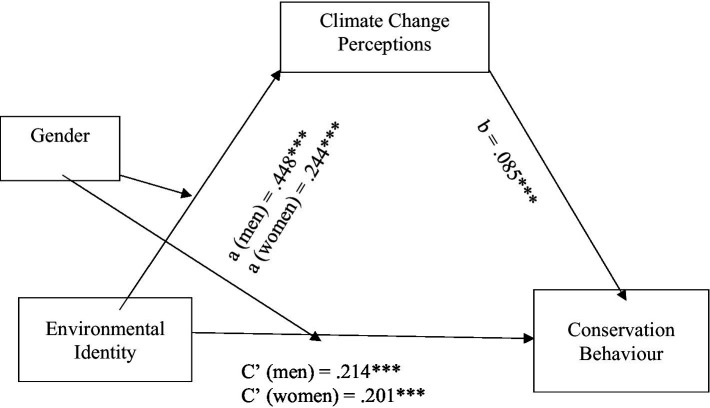
Conditional indirect effects of men’s and women’s environmental identity on conservation behaviour through climate change perceptions. ***p < 0.001.

**Figure 9:**
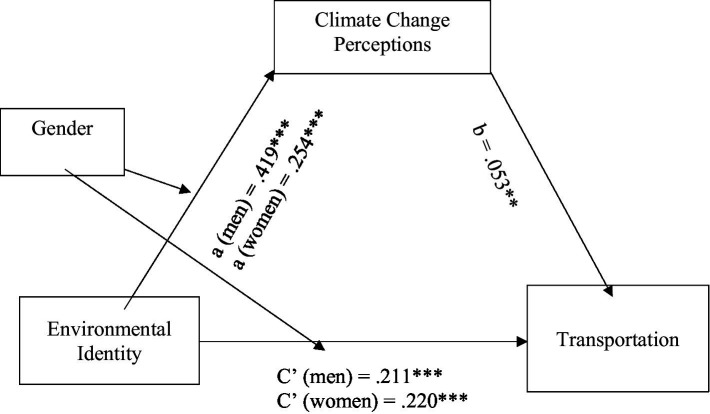
Conditional indirect effects of men’s and women’s environmental identity on transportation through climate change perceptions. **p < 0.01; ***p < 0.001.

**Figure 10:**
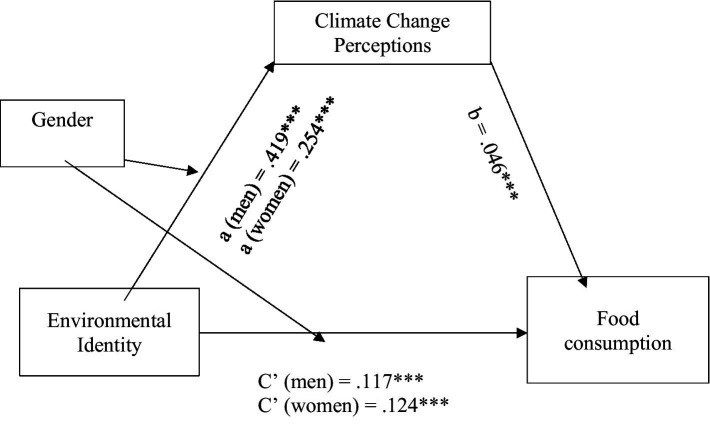
Conditional indirect effects of men’s and women’s environmental identity on food consumption through climate change perceptions. ***p < 0.001.

The last set of hypotheses suggested that the effect of environmental identity on climate change anxiety and pro-environmental behaviour would be mediated by climate change perceptions (Hypothesis 5), and that this mediation effect would be moderated by gender (Hypothesis 6). To test these hypotheses, model 8 of the PROCESS program (Hayes, 2022) was used to assess four moderated mediation models. In each of these models, climate change anxiety and the three measures of pro-environmental behaviour were the outcome variables and environmental identity was the predictor variable, with climate change perceptions as the mediator and gender as the moderator. Table 4 shows bias-corrected bootstrap estimates and 95% confidence intervals of the indirect (mediated) effects and the overall moderated mediation model. Figures 7–10 illustrate the estimates of the separate paths for women and men for each of the four outcome variables: climate change anxiety, conservation, transportation and food consumption behaviours. Partially consistent with hypothesis 5, climate change perceptions were a significant mediator in three of the models, as shown by the significant conditional indirect effects of environmental identity on pro-environmental behaviours for women and men. The results indicated that the effects of women’s and men’s environmental identities on their pro-environmental behaviours were mediated through their climate change perceptions, as the bootstrap confidence intervals for these effects were entirely above zero (Table 4). Nonetheless, the findings do not support the total conditional indirect effect of environmental identity on climate change anxiety through climate change perceptions for men or women, as the index of moderated mediation was not significant (Table 4). Interestingly, the direct effects of environmental identity on climate change anxiety were stronger for women than men (Figure 7, path c’).

**Table 4:** Bias-corrected bootstrap estimates for mediation and moderated mediation analyses.

Mediation by climate change perceptions									
	Women			Men			Moderated mediation		
	Estimate	95% CI		Estimate	95% CI		Estimate	95% CI	
		Lower	Upper		Lower	Upper		Lower	Upper
Climate change anxiety	0.006	−0.001	0.012	0.010	−0.001	0.020	−0.004	−0.009	0.001
Conservation behaviour	0.021***	0.014	0.029	0.038***	0.026	0.051	−0.017***	−0.027	−0.009
Transportation	0.013***	0.004	0.023	0.022***	0.014	0.029	−0.009***	−0.017	−0.002
Food consumption	0.012***	0.007	0.017	0.019***	0.012	0.028	−0.008***	−0.013	−0.003

***p < 0.001.

Results presented in Table 4 further illustrate that gender moderated this mediation effect of climate change perceptions, as indexes of moderated mediation were negative with bootstrap confidence intervals entirely below zero for conservation behaviour [−0.027, −0.009], transportation [−0.017, −0.002] and food consumption [−0.013, −0.003], as partially suggested by hypothesis 6. However, the total conditional indirect effects found were stronger for men than women, the opposite of what was expected. This meant that the indirect effect of environmental identity on the three pro-environmental behaviours examined through climate change perceptions was stronger for men than for women. On the other hand, the direct effects of environmental identity on transportation and consumption of vegetarian meals, Figures 9, 10 (paths c’) show that women’s environmental identity had a stronger direct positive effect on women’s use of alternative modes of transportation and consumption of vegetarian meals than men’s.

## 4 Discussion

This study was designed to explore how climate change anxiety and pro-environmental behaviour vary with gender and social psychological characteristics. Given the inconsistent evidence regarding gender differences in climate change anxiety and pro-environmental behaviour (e.g., Asgarizadeh et al., 2023; Clayton et al., 2023; Elert and Lundin, 2022; Islam and Managi, 2019), this study aimed to examine a nationally representative Portuguese sample and explore the complex social psychological mechanisms underneath, to clarify these inconsistencies.

The results revealed that climate change anxiety was positively related to the use of carpooling or alternative means of transportation and the consumption of vegetarian meals for men and women. Climate change anxiety was positively associated only with men’s conservation behaviour (e.g., reducing consumption of heating, air-conditioning). Taken together, these findings partially corroborate previous research (e.g., Hogg et al., 2021; Wullenkord et al., 2021). Moreover, for both men and women, climate change anxiety was positively related with environmental identity and climate change perceptions, consistent with previous research (e.g., Clayton and Karzsia, 2020; Galway et al., 2021).

Overall, the findings supported hypotheses 1 to 4. Specifically, the results revealed that women reported higher levels of climate change anxiety compared to men, and this was driven by women’s higher levels of climate change anxiety cognitive impairment. Additionally, women indicated more frequent conservation behaviours (e.g., reduce consumption of heating, air-conditioning, hot water, and lighting), more frequent consumption of vegetarian meals and use of alternative modes of transportation than men. These results extend the literature on gender differences in experiencing climate change anxiety and pro-environmental behaviour (e.g., Elert and Lundin, 2022; Heeren et al., 2022; Hickman et al., 2021; Maartensson and Loi, 2021; Wullenkord et al., 2021). The results can possibly be explained by the fact that women rely on domestic energy to a higher extent than men, as they are usually responsible for the lion’s share of housework and unpaid care (e.g., EIGE, 2024). On average, they also own fewer private vehicles and are more dependent on public transport than men (e.g., Cresswell, 2016; Cristaldi, 2005; EIGE, 2024).

Women also indicated higher levels of environmental identity and climate change perceptions than men, extending the findings of previous studies (e.g., Effrosynidis et al., 2022; McCright and Dunlap, 2011) and providing evidence of the role of gender in environmental identity and perceptions of climate change.

Environmental identity and climate change perceptions predicted higher levels of climate change anxiety and pro-environmental behaviours related to conservation and food for both men and women, echoing previous findings (e.g., Brügger et al., 2021; Clayton and Karzsia, 2020; Steg, 2023). Demonstrating, therefore, similar positive relations for men and women, between social psychological mechanisms (environmental identity and climate change perceptions) and their impact on pro-environmental behaviour. Nonetheless, climate change perceptions were only a significant predictor for women’s use of alternative modes of transportation.

The results further supported the predictions that climate change perceptions would mediate the effect of environmental identity on pro-environmental behaviours (partially sustaining Hypothesis 5), and these mediations were further moderated by gender (partially supporting Hypothesis 6). This meant that, compared to women, men’s climate change perceptions played a bigger part in the indirect effect of their environmental identity on their display of pro-environmental behaviour. On the other hand, for women, their environmental identity had a stronger direct positive effect on their use of alternative modes of transportation and consumption of vegetarian meals than men.

Regarding the experience of climate change anxiety, the findings did not support the total conditional indirect effect of environmental identity through climate change perceptions for men or women. However, the direct effects of environmental identity seemed more closely tied to women’s higher levels of climate change anxiety than men’s.

Climate change perceptions do not impact men’s and women’s environmental identity in their experience of climate change anxiety. However, environmental identity does impact women more directly in their experience of climate change anxiety than men.

Taken together, these results deepen our understanding of how environmental identity and climate change perceptions impact different types of pro-environmental behaviour and shed light on gender differences in climate change anxiety and its underlying drivers. It further highlights the importance of exploring the gender gap in environmental related attitudes and behaviours and the incorporation of gender mainstreaming in environmental sustainability policies and programmes.

The current study strengthens the accumulating evidence of the relevant role played by gender in experiencing climate change anxiety and enacting pro-environmental behaviours (Elert and Lundin, 2022; Heeren et al., 2022; Wullenkord et al., 2021) and how the underlying mechanisms can differ for men and women. It calls for the enhancement of the representation of women in environmental planning and the inclusion of gender equality objectives into environmental policies, with monitoring and evaluation mechanisms to assess progress. The design and implementation of gender-responsive policies and programmes prevent the reproduction of existing normative constructions of gender.

Gender mainstreaming is also important in the development of educational programmes addressing climate change anxiety. Mental health professionals should understand the gender gap and leverage social-psychological characteristics to increase pro-environmental behaviour.

Finally, future research should take an intersectional approach, accounting for other personal characteristics (e.g., race and ethnicity, disability, sexual orientation, and class) that contribute to unique experiences of climate change anxiety.

## 5 Limitations and future research

A few limitations should be considered when interpreting the results. First, the use of Clayton and Karzsia (2020) *Climate Anxiety-Scale* could represent a limitation as it does not take a comprehensive approach to the concept, focusing on impairment and not measuring other elements of climate change anxiety (e.g., van Dijk et al., 2025; Wullenkord et al., 2024). Consequently, a more comprehensive and multidimensional conceptualization of climate change anxiety should be taken in future research (Boehme et al., 2024).

Additionally, the measures used were based on self-reports, representing another limitation as they are subject to social desirability concerns and reduced reliability. Future research would benefit from integrating a combination of multiple sources of data (e.g., experimental designs, interviews) to evaluate climate change anxiety and pro-environmental behaviour.

Another limitation is the fact that the current study focuses exclusively on environmental identity and climate change perceptions, not exploring the relationships of other relevant socio-psychological variables (e.g., environmental knowledge, attitudes, values, social norms, affective factors) on climate change anxiety and pro-environmental behaviour.

The effect sizes in the findings are quite small, requiring a certain amount of caution when considering them. Nonetheless, they should not be dismissed, as it is a typical occurrence when investigating complex psychological processes (Götz et al., 2022).

Due to the cross-sectional nature of the study, no definitive causal conclusions can be made between gender, social psychological characteristics (environmental identities and climate change perceptions), climate change anxiety, and pro-environmental. Therefore, future studies would gain from using a longitudinal design, allowing for a better understanding of how gender and identities influence, and are influenced by climate change anxiety and pro-environmental behaviour.

In conclusion, the findings from the current study shed light on the role of gender in climate change anxiety and pro-environmental behaviour. Inclusive climate policies have the potential to drive social change and to address health and gender inequalities. Therefore, by understanding and addressing the gender gap in environmental policies and initiatives, societal change towards greater gender equality and green justice can be advanced (Echavarren, 2023).

## Data Availability

The datasets presented in this article are not readily available because of privacy reasons. Requests to access the datasets should be directed to the corresponding author.
